# A questionnaire study on the impact on oral health-related quality of life by conventional rehabilitation of edentulous patient

**DOI:** 10.1038/s41405-020-0029-5

**Published:** 2020-01-30

**Authors:** Bidhan Shrestha, Bishal Babu Basnet, Galav Adhikari

**Affiliations:** 1 Kantipur Dental College and Research Center, Kathmandu, Nepal; 2 BP Koirala Institute of Health Science, Dharan, Nepal; 3 Nepalese Army Institute of Health Sciences, Kathmandu, Nepal

**Keywords:** Prosthetic dentistry, Dentistry

## Abstract

**Purpose:**

This study aimed to determine whether complete dentures improve the oral health-related quality of life (OHRQoL) of edentulous patients, and to assess any associations related to age, gender, and OHRQoL.

**Material and methods:**

Hundred edentulous patients who required conventional complete denture treatment were selected for this study. The following inclusion criteria were established: edentulous in both jaws with no previous history of denture treatment and no significant medical history. The patients were selected who satisfied the criteria associated with the class I prosthodoctic diagnostic index. OHRQoL of the patients were assessed twice, once pre treatment (at the first visit) and once post treatment (8 weeks post insertion of dentures) using an instrument called OHIP-EDENT-N. Significant differences in the OHIP-EDENT-N scores between pre treatment and post treatment were calculated using the Wilcoxon Signed-Rank test. Gender differences were assessed using the Mann–Whitney test.

**Results:**

After provision of new complete dentures, all domains of the OHIP-EDENT-N showed significant improvements except physical pain and social disability.

**Conclusions:**

The results of this study indicated that conventional complete denture improved the OHRQoL of edentulous patients.

## Introduction

Health has been defined as “a state of complete physical, mental, and social well-being, not merely the absence of disease or infirmity” by the World Health Organization. Despite the relatively recent emergence of oral health-related quality of life (OHRQoL), its implications for the clinical practice of dentistry and dental research cannot be undermined. OHRQoL is an integral part of general health and well-being and is recognized by the WHO as an important segment of the Global Oral Health Program.^1^OHRQoL is a relatively new but rapidly growing phenomenon which has emerged over the past two decades.^2^ Several authors have explored the evolution of OHRQoL and documented the circumstances that have led to its prominence.^2–4^ Slade et al.^3–5^ identified that the shift in the perception of health, from merely the absence of disease and infirmity to the complete physical, mental, and social well-being is the key issue in the conception of HRQoL and subsequently OHRQoL.

OHRQoL is a multidimensional idea which can be defined as a person’s assessment of how functional, psychological, social factors, pain, or discomfort affect his/her well-being in the context of oral health.^6^ Tooth loss has a direct impact on normal functional activities in edentulous patients.^7^ There is a tendency of increase in the number of edentulous patients in almost every part of the world.^8^ This can be explained with the increase in life expectancy rate.^9–11^

Conventional complete dentures are the most widely used option for the rehabilitation of edentulous patients.^12^ Outcomes in prosthodontic therapy have focused mainly on the superior results of mandibular overdentures or fixed prosthesis as opposed to conventional complete denture.^13–15^ Compared with other treatment options, conventional complete dentures are relatively economical, esthetically acceptable, and cleansable.^16,17^ The physiological functions, otherwise lost due to edentulism, can be recovered within a short period of time with these conventional dentures.^13,18^

The Oral Health Impact Profile (OHIP) questionnaire is one of the most technically sophisticated instruments used for assessment of OHRQoL.^19^ The OHIP was developed and validated by Slade and Spencer^20^ and several versions of the tool have been developed thereafter. However, the tool comprises of 49 questions, which are divided into seven subscales, which made it comprehensive and arduous at the same time. The need for its short version was thus felt without affecting its scope of application. Among the available short versions, the OHIP-EDENT has been deemed the most appropriate tool for the edentulous patients, as it presented a set of specific questions. The tool detects the impact of oral health on the quality of life of patients with complete denture prostheses, before and after they have received them.^21^ Subsequently, to measure the oral health problems of geriatric patients, Geriatric Oral Health Assessment Index was developed by Atchison and Dolan.^22^

The purpose of this study was to determine whether complete dentures improved the OHRQoL of edentulous patients, and to assess the associations among age, gender, and OHRQoL in a select group of patients.

## Material and methods

After obtaining the ethical clearance from institutional ethical review board of BPKIHS (Acd/10/071/072) for the study, 100 completely edentulous patients planned for conventional complete denture treatment who fulfilled the following inclusion criteria: complete edentulousness with no previous history of denture treatment and no significant medical history. Further, patients who satisfied the criteria pertaining to Class I Prosthodontic Diagnostic Index were included in the study. Accordingly, the patients had mandibular bone height >21 mm, maxillary ridge morphology capable of resisting vertical and horizontal forces, adequate attached mucosa, and Class I maxillomandibular relationship. The patients were interviewed at pre treatment (at the first visit) and post treatment (at 8 weeks). Each session of the interview was of ~15 min and was conducted in the clinical area by a single interviewer at both visits to minimize the variability.

Exclusion criteria included:

- Patients requesting implant—supported prosthesis.
- Patients without a contact number as the patients had to be recalled for follow up by telephone.
- Patients having poor residual ridge, single complete dentures, temporomandibular joint problems, and psychiatric disorders.

The excluded patients underwent routine prosthodontic treatment. Written informed consent was obtained from the patients and they were assured that the research worker was not involved in their treatment and that their participation would not influence the outcome or cost of their treatment. OHRQoL of the patient was assessed using an instrument called OHIP-EDENT-N.^23^ The OHIP-EDENT-N is a 19-question survey in Nepalese version with seven subscales: functional limitation, physical pain, psychological discomfort, physical disability, psychological disability, social disability, and handicap. This tool aids in detecting impact of oral health on the quality of life of patients who wear prostheses.^23,24^ It is specific to edentulous patients and present questions addressing masticatory capacity, pleasure in eating, level of comfort and assuredness while wearing the prosthesis, and relationship problems among others. The tool detects the impact of oral health on the quality of life of patients with prostheses, before and after they have received them.^21^ The item impact method is used to select items that are most relevant to edentulous patients. The questionnaire gives a choice of five answers. A simple score was calculated by adding the responses to all the questions (0 = never; 1 = seldom; 2 = fairly often; 3 = often; 4 = very often). It ranges from 0 to 76. The lowest score represent a satisfactory perception of an individual’s oral conditions, and therefore higher satisfaction and better quality of life. This instrument is reliable and valid in Nepalese version.^23^ After the initial interview, complete dentures, in balanced occlusion, were fabricated for all the patients by postgraduate students as per the standard norms being followed in the Department of Prosthodontics, BPKIHS. Post insertion adjustments were made as needed for the patients. After the patients found their dentures acceptable and did not report with any further complaints, a 5-week time interval was set and the second interview was then held. The data obtained were entered into a Microsoft Excel 2010 for windows (Microsoft Corp., Redmond, WA) spreadsheet. Shapiro–Wilk tests were performed for testing the normality. Since the assessments were done based on scores, and the scores were not normally distributed, nonparametric methods were used for the analysis. Standard descriptive statistical calculations were computed for all the variables at pre treatment and after 8 weeks. Significant differences in the OHIP-EDENT-N scores between pre treatment and post treatment were calculated using the Wilcoxon Signed-Rank test. Gender differences were assessed using the Mann–Whitney test. Statistical significance was set at *p* *<* 0.05 and *p* *<* 0.001. All analyses were carried out using statistical package for social sciences version 20 for windows (IBM SPSS statistics, Chicago, IL).

## Results

A total of 100 patients agreed to participate in the study. The study was carried out for a year (2014 July–2015 July). At the post treatment interview, six participants did not attend the second interview and six participants were not wearing dentures at all. Hence, a final sample of 88 participants (59 men and 29 women, median age 67 years, range 53–91 years) were included in the study. Participants with age ≤65 years reported slightly higher impact on all domains compared with participants with age above 65 years. The highest scores were recorded in physical disability (Mean = 4.8, SD = 3.4, Median = 3.88) and functional limitation (Mean = 4.3, SD = 3.6, Median = 4.2). In participants over the age of 65, the highest impacts were recorded in the domains physical disability, physical pain, and functional limitations. However, significant relationship was not observed between age and OHRQoL. Although females recorded higher impacts on all domains, the relationship between gender and OHRQoL was not statistically significant. Table 1 shows the comparison between frequency distribution of OHIP-EDENT-N scores before and after treatment. The items with high impact pre treatment included difficulty in chewing foods and avoiding some foods, uncomfortable to eat with the dentures, and being self-conscious. The items that occurred substantially less often post treatment included being self-conscious because of teeth, mouth or dentures, being worried about dental problems, avoiding certain foods and being upset or embarrassed because of problems with teeth, mouth, or dentures.

**Table 1:** The frequency distributions of the scores of the OHIP-EDENT-N pre treatment and post treatment.

Domain	OHIP-EDENT-N question (Baseline)	Frequency % Combined*234	
		Pre treatment	Post treatment
FL	1. Have you had difficulty chewing any foods because of problems with your teeth, mouth or dentures?	81.8	53.4
FL	2. Have you had food catching in your teeth or dentures?	18.2	14.8
P1	3. Have you had painful aching in your mouth?	11.4	29.5
P1	4. Have you found it uncomfortable to eat any foods because of problems with your teeth, mouth or dentures?	79.5	43.2
P1	5. Have you had sore spots in your mouth?	8	14.8
FL	6. Have felt that your dentures have not been fitting properly?	19.3	42
P1	7. Have you had uncomfortable dentures?	18.2	30.7
P2	8. Have you been worried by dental problems?	48.9	22.7
P2	9. Have you been self-conscious because of your teeth, mouth or dentures?	78.4	29.5
D1	10. Have you had to avoid eating some foods because of problems with your teeth, mouth or dentures?	80.7	63.6
D1	11. Have you been unable to eat with your dentures because of problems with them?	43.2	40.9
D1	12 Have you had to interrupt meals because of problems with your teeth, mouth or dentures?	33	25
D2	13. Have you been upset because of problems with your teeth, mouth or dentures?	50	21.6
D2	14. Have you been a bit embarrassed because of problems with your teeth, mouth or dentures?	48.9	6.8
D3	15. Have you avoided going out because of problems with your teeth, mouth or dentures?	50	3.4
D3	16. Have you been less tolerant of your partner or family because of problems with your teeth, mouth or dentures?	13.6	6.8
D3	17. Have you been irritable with other people because of problems with your teeth, mouth or dentures?	18.2	4.5
H	18. Have you been unable to enjoy other peoples company as much because of problems with your teeth, mouth or dentures?	55.7	1.1
H	19. Have you felt that life in general was less satisfying because of problems with your teeth, mouth or dentures?	87.5	29.5

*FL* functional limitation, *P1* physical pain, *P2* psychological discomfort, *D1* physical disability, *D2* psychological disability, *D3* social disability, *H* handicap.

*2, 3, 4 = occasionally, fairly often and very often.

The impact of a few items was greater in post treatment, such as painful aching, denture not fitting properly, sore spots in the mouth, and uncomfortable dentures.

Table 2 shows that the participants reported less OHRQoL impacts following the receipt of their new dentures in all domains except *physical pain* and *social disability*. Significant relationships were recorded in all the domains (*p* < 0.001) except physical pain and social disability. The summary score reflected an improvement of with regards to OHRQoL.

**Table 2:** Relationship between OHRQoL (pre treatment) and OHRQoL (post treatment).

Domains	OHIP-EDENT-N Scores		Paired mean difference	*p*^a^ value
	Baseline mean ± SD	Post mean ± SD		
Functional limitation	5.25 ± 3.27	4.01 ± 3.39	1.23	0.001
Physical pain	3.67 ± 2.78	4.01 ± 3.39	−0.34	0.8
Psychological discomfort	7.64 ± 5.06	3.43 ± 4.09	4.21	0.001
Physical disability	5.72 ± 2.97	4.35 ± 3.45	1.37	0.001
Psychological disability	4.57 ± 4.17	1.01 ± 1.64	3.55	0.001
Social disability	0.14 ± 0.61	0.14 ± 0.61	0	1
Handicap	7.14 ± 4.07	2.04 ± 1.93	5.09	0.001
Overall	34.19 ± 16.6	19 ± 15.99	15.27	0.001

*p* < 0.05 denotes significance; *p* < 0.001 denotes high significance.

^a^Wilcoxon Signed-Rank test.

## Discussion

It was found that completely edentulous patients have a considerably impaired level of OHRQoL prior to treatment. However, as indicated by reduced mean OHIP-EDENT-N scores, the OHRQoL improved within 5 weeks following complete denture treatment. A statistically significant improvement was observed in domains like functional limitation, psychological discomfort, psychological disability, physical disability, and handicap. However, new complete dentures did not contribute greatly to improved mastication or the relief of denture-related pain.

The majority of the study subjects were males (67%). Many other studies have also shown significant gender difference in the edentulism with more males becoming edentulous than females.^25,26^ This has been attributed to the fact that males have greater number of parafunctional oral habits like tobacco, betel nut, pan chewing, and smoking compared with females and don’t pay much attention to oral care. However, other studies have reported edentulism being more common in females than in males.^27^

As reported by John et al. and Heydecke at al., this study also found that younger patients reported more OHRQoL impacts.^28,29^ This implies that the impact of oral diseases decreases with age. This may also be because we find older patients more likely to be more accepting of their fate. They also feel that these are problems associated with aging and a part of life.

In this study females showed a higher impact in OHRQoL. This is supported by Mersel et al. who found that male patients are often more satisfied with their dentures regarding comfort, function, and appearance.^30^

The paired mean differences showed positive improvements across all domains except *physical pain* and social disability. However, these small negative results could be attributed to the new complete dentures. Some patients were experiencing post treatment pain, which subsequently affected their normal eating. The OHIP-EDENT-N summary scores indicated an improvement of 15 units. The findings of this study showed an improvement in the initial scores after complete denture rehabilitation as found in other studies.^14,29,31^ However, the magnitude of improvement in OHRQoL observed here was not as great as reported in other literature. Differences in patient populations, time period of evaluation, and sampling variability may be responsible. Despite the favorable outcomes, conventional complete denture patients have still been reported to have a poorer OHRQoL than implant patient groups.^13,32^

The summary scores before and after prosthodontics treatment in our study were lower than those reported in a study conducted by John et al.^29^ when mean OHIP-G-49 values were compared. While this may indicate that the patients in this study have a poorer overall OHRQoL than the German patients, results from both the studies are consistent in demonstrating improvements in OHRQoL following prosthodontic treatment. The findings of this study support the belief that patients benefit from properly fitting dentures. Garret et al.^33^ reported similar findings, where almost all the patients perceived improvement in masticatory function, speech, and security.^33^ The results of this study also suggest that the OHIP-EDENT-N is able to detect oral health changes over time and to measure the effects of oral treatments. This is in accordance with previous longitudinal studies where patients who benefited from the placement of new dentures saw their quality of life improved.^18^

One of the limitation of the study is small sample size. The other limitation is lack of regular follow up due to poor compliance of the patients, thus some of the patients were having pain even after 5–6 weeks of CD delivery.

## Conclusions

The findings of this study have provided an overview of impaired OHRQoL in complete denture wearers in the eastern region of Nepal.

The analyses of these results provided evidence for the following:

1. The study showed that complete dentures improved the OHRQoL of patients. Significant improvements were recorded in most domains except physical pain and social disability. These results are relevant for clinicians in drawing evidence about the benefits of treatment when advising patients about whether treatment will improve their oral function and everyday lives.
2. Age and gender had a weak relationship with OHRQoL. However, the small number of female participants in the study may be considered as a limitation as a clearer relationship between gender and OHRQoL could not be drawn.

## Data Availability

Data will be made available on request.
